# 

**Published:** 1801-03

**Authors:** 


### TO CORRESPONDENTS.

*The original Communications, as usual at this time of the year, have been so numerous, that we have been compelled to postpone the insertion of several valuable Papers, Accounts of Books, Hints, &c.*

